# Biomarkers of early sepsis may be correlated with outcome

**DOI:** 10.1186/1479-5876-12-146

**Published:** 2014-05-26

**Authors:** Tsai-Hsia Hong, Chin-Hao Chang, Wen-Je Ko, Ching-Feng Lin, Heng-Hsiu Liu, Lu-Ping Chow, Chun-Ta Huang, Sun-Liang Yu, Yih-Sharng Chen

**Affiliations:** 1 Department of Surgery, National Taiwan University Hospital, Taipei 100, Taiwan; 2 Department of Medical Research, National Taiwan University Hospital, Taipei, Taiwan; 3 Department of Traumatology, National Taiwan University Hospital, Taipei, Taiwan; 4 Institute of Epidemiology and Preventive Medicine, College of Public Health, National Taiwan University, Taipei, Taiwan; 5 Department of Biochemistry, College of Medicine, National Taiwan University, Taipei, Taiwan; 6 Department of Clinical Laboratory Sciences and Medical Biotechnology, College of Medicine, National Taiwan University, Taipei, Taiwan

**Keywords:** MCP-1, IL-6, IL-8, IL-10, Severe sepsis

## Abstract

**Background:**

Sepsis causes high mortality, and the mortality due to secondary infections is even higher. No studies to date have investigated the time from the primary infection to death due to a secondary infection; similarly, the factors that are significantly different in sepsis survivors relative to non-survivors or in severe sepsis patients who suffered a late death relative to those who recover have not been explored. We hypothesized that patients who survive sepsis have a weaker pro-inflammatory response than those who do not and that the mid-term survivors (which acquire secondary infections) would have a pronounced anti-inflammatory response (making them susceptible to infection); this hypothesis was verified in this study.

**Methods:**

We examined 24 patients with severe sepsis; the patients were subdivided by outcome into early death (n = 5), mid-term survival (survival through severe sepsis but death within six months or continued hospitalization for six months, n = 6), and long-term survival (recovery and survival for more than six months, n = 13) groups. The levels of CD3^+^, CD4^+^, CD8^+^, and CD19^+^ lymphocytes were analyzed by flow cytometry, and the plasma levels of carbonic anhydrase IX (CA IX), MCP-1, IL-6, IL-7, IL-8, and IL-10 were measured by ELISA on days 0, 1, 2, and 3. A statistical comparison of the variables in the groups was conducted using a mixed model.

**Results:**

The plasma levels of MCP-1, IL-6, and IL-8 in early death and survivors were significantly different, and all had *p* values <0.01. The plasma levels of MCP-1, IL-6, and IL-8 were also significantly different in mid-term survivors and long-term survivors, with *p* values of <0.01, 0.04, and <0.01, respectively.

**Conclusions:**

Our data support the hypothesis that survivors have a weaker pro-inflammatory response than non-survivors, but the mid-term survivors did not have a more pronounced anti-inflammatory response. The levels of pro-inflammatory cytokines in the mid-term and long-term survivors were significantly different.

## Background

Sepsis is one of main causes of death in intensive care units (ICUs), and estimates of its prevalence range from 25% to 70% [1-5]. Sepsis often exhibits two stages concomitantly; systemic inflammatory response syndrome (SIRS) releases inflammatory cytokines, and the compensatory anti-inflammatory system (CARS) raises the concentration of anti-inflammatory mediators [6,7]. This overwhelming inflammatory response leads to early mortality and tilts the precise balance of inflammation and anti-inflammation towards recovery, and excessive anti-inflammation often leads to secondary infections [6,8]. The latter is known as immunoparalysis [9]. Cytokine production precedes the expansion of CD14^+^CD16^+^ monocytes [10]. The levels of IL-8, IL-6, and monocyte chemoattractant protein-1 (MCP-1) are associated with early 48-hr and 28-day mortality in sepsis patients [11]. The duration of immunoparalysis is not known; therefore, sepsis management strategies do not differ for patients with the late death or recovery outcomes because prognostic markers for the early stages are not available. However, if these outcomes could be predicted at an early stage, patient management could be tailored to reduce late mortality.

We hypothesized that survivors would have a weaker pro-inflammatory response than non-survivors, while mid-term survivors (which acquire secondary infections) would have a more pronounced anti-inflammatory response (making them susceptible to infection). Therefore, the plasma levels of cytokines and lymphocyte subpopulations from cases of early death, mid-term survivors, and long-term survivors were measured in the early phases of severe sepsis to verify this hypothesis.

## Methods

### Study population

This study was approved by the Research Review Committee of National Taiwan University Hospital (NTUH). The enrolled severe sepsis patients were admitted to our institution between December 10, 2010 and December 10, 2011 and had a highly probable or proven infection and at least three of the following systemic inflammatory response syndrome (SIRS) criteria: body temperature >38°C or <36°C; heart rate >90/min; breathing frequency ≥20/min, PaCO_2_ < 32 mmHg, or ventilator use; leukocyte count >12000/mm^3^ or <4000/mm^3^ or >10% band forms; acute altered mental status; hyperglycemia without a history of diabetes; or blood glucose > 120 mg/dL. Other inclusion criteria were age ≥18 years, admission to the ICU, and at least one organ failure due to sepsis or septic shock. Septic shock was defined as sepsis with hypotension refractory to fluid challenge. Pregnant women, patients who had refused resuscitation, those with known or suspected human immunodeficiency virus infection, and those with known or suspected underlying immune deficiency were excluded. The zero time point was designated as being within 12 hours after the first organ failure due to sepsis. Blood was collected at the zero time point (day 0) and on days 1, 2, and 3.

### Flow cytometric analysis

Blood was collected into a Vacutainer tube containing EDTA. One hundred microliters of blood was stained with CD3 PerCP/CD19 APC/CD4 FITC/CD 8PE (Becton Dickinson, San Jose, California, USA) at room temperature for 25 minutes in the dark, lysed with BD lysing solution, washed two times with phosphate buffer saline (PBS) containing 1% heat-inactivated fetal bovine serum, and fixed with PBS containing 0.25% paraformaldehyde. For each test, 20,000 leukocytes were collected and analyzed using a BD FACSCalibur flow cytometer with CellQuest software version 3.2 (Becton Dickinson, San Jose, California, USA).

### Cytokine, chemokine, and carbonic anhydrase (CA) IX analysis

Blood was collected in a sodium heparin vacutainer tube. Plasma was collected, aliquot, and stored at −80°C until analysis. The plasma concentrations of MCP-1, IL-6, IL-7, IL-8, and IL-10 were separately analyzed by commercial ELISA kits according to the corresponding manufacturers’ instructions. The MCP-1 kit was obtained from eBioscience (San Diego, California, USA), the IL-7 kit was obtained from BioLegend (San Diego, California, USA), and the other cytokine kits were obtained from Becton Dickinson. Carbonic anhydrase (CA) IX is considered to be a marker of hypoxia [12] and was therefore included in the study. The CA IX kit was obtained from Oncogene Science (Cambridge, Massachusetts, USA).

### Statistical Analysis

The patients were divided into the death or survival groups according to outcome. The patients in the survival group were further divided into a mid-term survival group (MTSG, characterized by survival through severe sepsis but death within six months or continued hospitalization for six months) and long-term survival group (LTSG, characterized by recovery from sepsis with survival for more than six months). The groups were evaluated at 4 different time points (Day 0, 1, 2, 3) for ten biomarkers (CA IX, MCP-1, IL-6, IL-7, IL-8, and IL-10, as well as the percentages of CD3^+^, CD4^+^, CD8^+^, and CD19^+^ lymphocytes). The biomarker levels were LOG10 transformed in all analyses.

The overall comparisons of the groups (the survival group versus the death group and LTSG versus MTSG) and time points for each biomarker were carried out using a mixed model. An appropriate covariance structure specification for each biomarker was chosen from four different covariance structures (unstructured (UN), Huynh-Feldt (HF), compound symmetry (CS), and first order autoregressive (AR1)) based on the smallest values of the Akaike Information Criteria (AIC) and Bayesian Information Criteria (BIC) [13]. The analysis of effects was carried out after the selection of a covariance structure. The *p*-values were calculated using Satterthwaite’s approximation in PRO MIXED. Values were determined to be significantly different when *p* < 0.05. All statistical analyses were performed in SAS version 9.2 (Cary, North Carolina, USA), and the above models were used.

## Results

### Characteristics of the Patients

**Figure 1:**
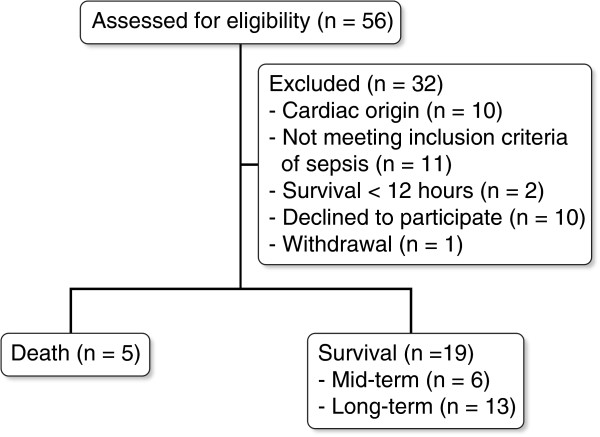
Flow chart of the cases included in this study.

During the study period, we screened 56 patients; 34 of these fulfilled the inclusion criteria. Ten patients withdrew informed consent. A flow chart depicting the enrolled patients is shown in Figure 1. Twenty-four cases were ultimately enrolled and diagnosed as having pneumonia, urinary infections, bed sore infections, cellulitis, and intra-abdominal abscesses. Five cases (20.8%) died of the sepsis episode separately on days 2, 5, 7, 10, and 10. The characteristics of the enrolled cases are given in Table 1. Among the MTSG patients, five patients separately died of a secondary infection on days 19, 44, 45, 49 and 176 due to sepsis/septic shock, and one was hospitalized due to repeated infections in six months. The patient with continued hospitalization for six months was diagnosed with necrotizing pancreatitis with persistent multiple intra-abdominal abscesses. This patient was transferred to the ICU of another hospital after a six-month hospitalization at NTUH. The patients in the recovery group were healthy upon discharge from the hospital in less than six months from admittance.

**Table 1:** Characteristics of the severe sepsis patients

**Diagnosis**	**Death (N = 5)**	**Survival**	
		**Mid-term (N = 6)**	**Long-term (N = 13)**
Median survival time (days)	6.46	45	NA
Female/male	1/4	1/5	9/4
Age (years)	72.0 ± 13.6	64.8 ± 13.5	78.0 ± 11.5
Pneumonia	2	4	5
*K. pneumoniae*	0	0	0
MSSA	0	0	1
MRSA	1	1	0
*H. influenzae*	0	1	0
*E. cloacae, K. pneumoniae*	1	0	0
*S. marcescens*	0	0	1
*E. coli*	0	1	0
Urinary tract infection	1	0	3
*K. pneumoniae*	1	0	0
*E. coli*	0	0	1
*E. coli, A. baumannii*	0	0	1
Bed sore infection	1	0	0
*M. morganii*	1	0	0
*C. albicans*	0	0	0
Cellulitis	0	0	1
Intra-abdominal abscess	1	2	4
*E. coli*	0	1	0
*A. baumannii, S. maltophilia*	0	1	0
*A. lwoffii*	0	0	1
*K. pneumonia*	1	0	0
Blood culture positive rate	100%	83.3%	38.5%

Death: cases who died due to sepsis; Mid-term survival: cases who survived sepsis but died or were in continued hospitalization within six months; Long-term survival: cases who had complete recovery and survived >6 months.

### The levels of CD3^+^, CD4^+^, CD8^+^, and CD19^+^ lymphocytes in the early death and survival groups are not significantly different

**Figure 2:**
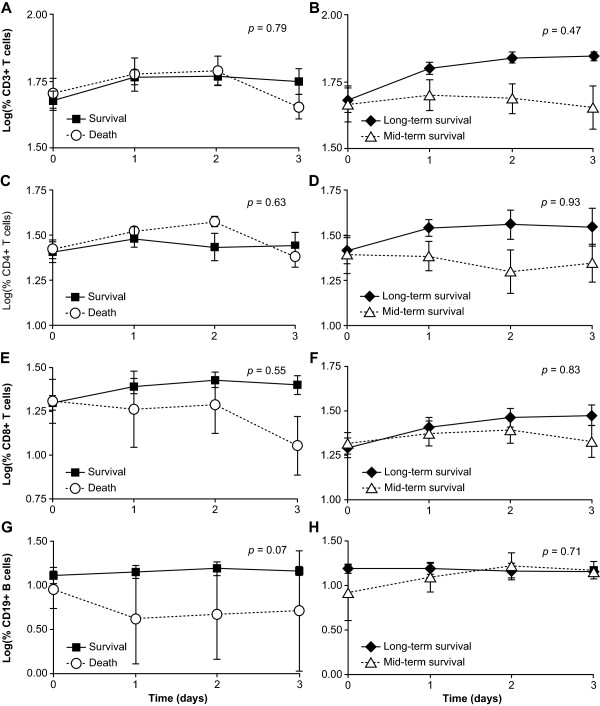
**Comparisons of lymphocyte subpopulations between the death and survival groups (2A, 2C, 2E, and 2G) and between the mid-term and long-term survival groups (2B, 2D, 2F, and 2H).** The mean ± standard error of each group at each time point are shown; the subpopulation frequencies are LOG10 transformed. The differences between the groups were considered to be significant when *p* < 0.05. CA: carbonic anhydrase.

The dynamic changes in the proportions of CD3^+^, CD4^+^, CD8^+^, CD19^+^ lymphocytes in the death and survival groups are shown in sections A, C, E, and G of Figure 2. A decrease in the CD3^+^, CD4^+^ and CD8^+^ lymphocyte levels was observed in the death group on day 3. Compared to the CD8^+^ lymphocyte level, the proportion of the CD4^+^ lymphocytes was lower in the death group on day 3. An overall comparison between these two groups revealed no significant differences using a mixed model, as shown in Table 2.

**Table 2:** **Comparisons of the percentages of CD3**^**+**^**, CD4**^**+**^**, CD8**^**+**^**, and CD19**^**+ **^**lymphocytes in the death and survival groups and the long-term survival group (LTSG) and mid-term survival group (MTSG) using a mixed model**

**Survival Group versus Death Group**					**LTSG versus MTSG**				
**Variable**		**Model-Based**	**Test**	***p***	**Variable**		**Model-Based**	**Test**	***p***
		Estimated Mean*(SE)					Estimated Mean*(SE)		
CD3^+^ T			0.08	0.79	CD3^+^ T			0.55	0.47
	Survival	1.72 (0.04)				LTSG	1.74 (0.04)		
	Death	1.76 (0.06)				MTSG	1.70 (0.05)		
CD4^+^ T			0.24	0.63	CD4^+^ T			0.01	0.93
	Survival	1.43 (0.05)				LTSG	1.44 (0.06)		
	Death	1.48 (0.09)				MTSG	1.45 (0.07)		
CD8^+^ T			0.36	0.55	CD8^+^ T			0.05	0.83
	Survival	1.34 (0.06)				LTSG	1.34 (0.05)		
	Death	1.27 (0.09)				MTSG	1.36 (0.07)		
CD19^+^ B cell			3.63	0.07	CD19^+^ B cell			0.14	0.71
	Survival	1.17 (0.12)				LTSG	1.15 (0.07)		
	Death	0.67 (0.24)				MTSG	1.18 (0.08)		

CD3^+^ T cells: the percentage of CD3^+^ T cells in lymphocytes; CD19^+^ B cells: the percentage of CD19^+^ B cells in lymphocytes.

### The levels of CD3^+^ and CD4^+^ lymphocytes in the mid-term survival group are lower than those in the long-term survival group

Comparisons of the lymphocyte subpopulations of the MTSG and LTSG are shown in sections B, D, F, and H of Figure 2. The levels of CD3^+^ and CD4^+^ lymphocytes were lower in the MTSG than in the LTSG. Although lymphocyte apoptosis has been reported in sepsis, the observation that there are lower levels of CD3^+^ and CD4^+^ lymphocytes in the MTSG is novel. The results of the mixed model are shown in Table 2. No significant differences were found between the lymphocyte subpopulations of the MTSG and LTSG.

### The levels of MCP-1, IL-6, and IL-8 in the early death and survival groups were significantly different

**Figure 3:**
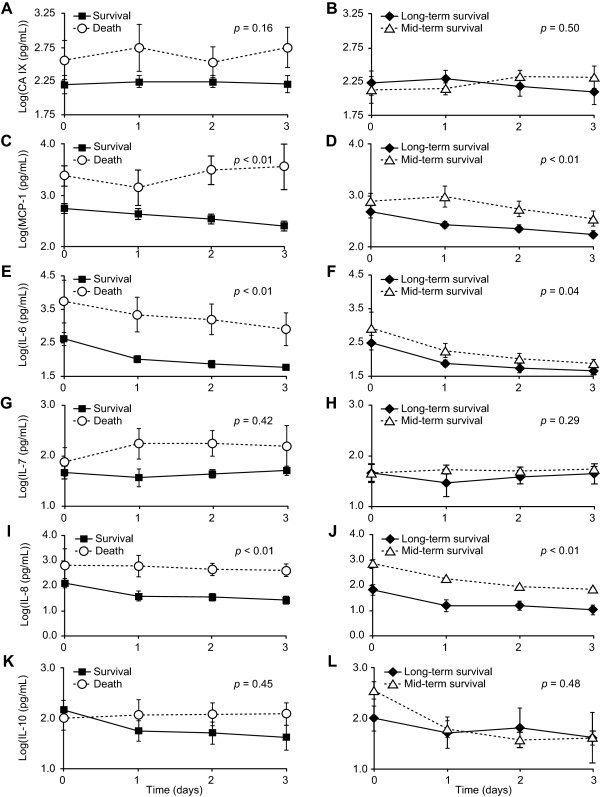
**Comparisons of CA IX, MCP-1, IL-6, IL-7, IL-8, and IL-10 between death and survival (3A, 3C, 3E, 3G, 3I, and 3K) and between mid-term survival and long-term survival groups (3B, 3D, 3F, 3H, 3J and 3L).** The mean ± standard error of each group at each time point are presented in LOG10 transformation. The difference between groups was considered significant with *p* < 0.05. CA: carbonic anhydrase, MCP: monocyte chemoattractant protein, IL: interleukin.

Comparisons of the CA IX, MCP-1, IL-6, IL-7, IL-8, and IL-10 levels in the death and survival groups are shown in sections A, C, E, G, I, and K of Figure 3. A statistical analysis of the overall difference between the groups is shown in Table 3. The plasma levels of MCP-1, IL-6, and IL-8 were significantly different in the death and survival groups, with *p*-values all less than 0.01. The plasma levels of IL-10 in the death group were higher than those of the survival group after day 0. Imbalanced levels of inflammatory and anti-inflammatory cytokines were present in the severe sepsis patients before death.

**Table 3:** Comparisons of the levels of CA IX, MCP-1, IL-6, IL-7, IL-8 and IL-10 in the death and survival groups and the long-term survival group (LTSG) and mid-term survival group (MTSG) using a mixed model

**Survival Group versus Death Group**					**LTSG versus MTSG**				
**Variable**		**Model-Based**	**Test**	***p***	**Variable**		**Model-Based**	**Test**	***p***
		Estimated Mean* (SE)					Estimated Mean* (SE)		
CA IX			2.13	0.16	CA IX			0.48	0.50
	Survival	2.26 (1.10)				LTSG	2.21 (0.10)		
	Death	2.51 (0.16)				MTSG	2.29 (0.12)		
MCP-1			18.97	<0.01	MCP-1			18.97	<0.01
	Survival	2.59 (0.09)				LTSG	2.56 (0.09)		
	Death	3.33 (0.17)				MTSG	3.38 (0.17)		
IL-6			11.9	<0.01	IL-6			6.51	0.04
	Survival	2.08 (0.14)				LTSG	1.96 (0.09)		
	Death	3.25 (0.23)				MTSG	2.23 (0.11)		
IL-7			0.71	0.42	IL-7			1.43	0.29
	Survival	1.67 (0.13)				LTSG	1.70 (0.15)		
	Death	1.86 (0.22)				MTSG	1.46 (0.19)		
IL-8			28.9	<0.01	IL-8			18.6	<0.01
	Survival	1.56 (0.15)				LTSG	1.26 (0.14)		
	Death	2.82 (0.23)				MTSG	2.15 (0.18)		
IL-10			0.59	0.45	IL-10			0.51	0.48
	Survival	1.76 (0.15)				LTSG	1.62 (0.21)		
	Death	2.02 (0.29)				MTSG	1.87 (0.29)		

CA IX: carbonic anhydrase IX; MCP-1: monocyte chemoattractant protein-1.

### The levels of IL-6, IL-8 and MCP-1 in the mid-term and long-term survival groups were significantly different

Comparisons of the plasma variables of the MTSG and LTSG are shown in sections B, D, F, H, J, and L of Figure 3. The overall levels of MCP-1 (*p* < 0.01), IL-6 (*p* = 0.04), and IL-8 (*p* < 0.01) in the mid-term and long-term survival groups were significantly different based on the mixed model.

## Discussion

The plasma levels of MCP-1, IL-6, and IL-8 in the early death and survival groups were significantly different during the early stages of the sepsis episode. The surviving patients recovered from this infection, while the others died from a secondary infection. The plasma levels of IL-6, IL-8, and MCP-1 in these two groups of patients were significantly different; this is a novel finding of the study. Our data support the hypothesis that sepsis survivors have a weaker pro-inflammatory response than non-survivors and reject the hypothesis that mid-term survivors have a more pronounced anti-inflammatory response. The duration of immunosuppression after SIRS is not known. One MTSG case died on day 176, and one case was hospitalized for more than six months. Therefore, the recovery group was designated as those patients who were healthy at the time of discharge from the hospital and lived longer than six months after sepsis.

A finely tuned balance between pro- and anti-inflammatory events is a prerequisite for better prognosis in sepsis. T-helper (Th)1 response predominates after microbial infection, which activates cells to directly clear an infection. Th2 cytokine secretion is then induced to resolve the pro-inflammatory response and to stimulate a humoral response. However, during sepsis, a Th2 response causes the dysregulation of the cellular immune response instead of resolving infection. Th2 cytokines inhibit the Th1 response, and vice versa. IL- 10, a potent anti-inflammatory cytokine, has a strong suppressive effect on monocytes/macrophages, dendritic cells, neutrophils and T cells [14]. In this study, the plasma levels of IL-10 were not significantly different in the early death group versus the survivors or in the MTSG versus the LTSG. Higher levels of IL-10 were present in the MTSG on day 0, which implies that the MTSG was experiencing immunosuppression. Although the levels of IL-6, IL-8, and MCP-1 in the MTSG and LTSG were significantly different in the early phase, the MTSG patients survived until the secondary infection. High levels of IL-8 and MCP-1 would attract, activate, and promote neutrophil and monocyte migration toward the site of inflammation, as well as to the remote organs. Excessive neutrophil and monocyte infiltration exaggerates inflammation and severe organ injury by releasing pro-inflammatory mediators, such as IL-6, which can lead to shock, multiple organ failure, and even death [15].

Our results indicate that IL-6, IL-8, and MCP-1 were much more potent than IL-10 in promoting death due to a secondary infection. Factors other than IL-10 may be more effective in inhibiting inflammation in the early phase of sepsis. IL-7 has anti-apoptotic properties and induces the proliferation of CD4^+^ and CD8^+^ naïve T cells [15]. Because lymphocyte apoptosis has been reported in sepsis, the level of IL-7 was expected to be higher in the survival group and the LTSG. However, the levels of IL-7 were higher in both the death group and the MTSG, which conflicts with our expectations. A further investigation of this effect is desired.

A distinctive drop in the proportion of CD3^+^ lymphocytes, particularly CD8^+^ lymphocytes in the death group on day 3, was observed in this study. The level of CD19^+^ B lymphocytes in the death group was elevated on day 3; this may be due to the decrease in the proportion of both CD4^+^ and CD8^+^ lymphocytes. CD8^+^ lymphocytes were most likely more fragile and eliminated more on day 3. These effects also require further investigation.

No comparisons of the lymphocyte subpopulations of patients who survive or died due to sepsis or between MTSG and LTSG were conducted in previous studies. In one example, the leukocyte subpopulations of 8 cases of pneumonia-derived sepsis (PDS) with a 37.5% survival rate and 14 cases of intra-abdominal sepsis (IAS) with a 35.7% survival rate were compared to those from normal controls over the course of 4–6 days after the onset of sepsis. Lymphocyte levels were diminished compared to controls in both types of sepsis, and a marked drop in CD3^+^CD8^+^ lymphocytes was observed [16]. Another study reported that the levels of CD3^+^CD4^+^ and CD19^+^ lymphocytes and the CD4^+^/CD8^+^ T cell ratio were significantly lower in 25 septic shock non-survivors (*P* < 0.01) compared to 27 survivors on the day of hospitalization; however, there was no difference in the proportion of CD3^+^CD8^+^ T lymphocytes between non-survivors and survivors [17]. A discrimination of the MTSG and LTSG was not conducted in their study. The zero time point was also restricted in our study, whereas previous authors defined day zero as the day of hospitalization; one drawback of this approach is that the onset of sepsis may not be clear. The number of cases included in this study was reduced due to our strict definition of the zero point to within 12 hours after the first organ failure due to sepsis. More cases will be needed to validate these results.

One of our inclusion criteria in the present study is that patients were recruited with 12 hours after the first organ failure due to sepsis as the zero time point. It caused that we were not able to recruit enough patients. Thus, the main limitation of the present study is the small sample size. In the future studies, more patients will be needed for validation.

The presence of high levels of MCP-1, IL-6, IL-8, and IL-10 in the death group in our study indicated an imbalance of inflammation and anti-inflammation; this imbalance promotes overwhelming inflammation, leading to death. The levels of MCP-1, IL-6, and IL-8 in the early death and survival groups and between the MTSG and LTSG were significantly different. Pro-inflammatory cytokines were more prominent in the MTSG.

## Conclusions

Our data support the hypothesis that sepsis survivors would have a weaker pro-inflammatory response than non-survivors, whereas mid-term survivors did not have a more pronounced anti-inflammatory response. The levels of pro-inflammatory cytokines were significantly different in mid-term and long-term survivors.

## Abbreviations

AUC: The area under the receiver operating characteristic curve; CA: Carbonic anhydrase; CARS: The compensatory anti-inflammatory system; IAS: Intra-abdominal sepsis; ICU: Intensive care unit; LTSG: The long-term survival group; MCP-1: Monocyte chemoattractant protein-1; MTSG: The mid-term survival group; NTUH: National Taiwan University Hospital; PDS: Pneumonia-derived sepsis; REC: Research Ethics Committee; SIRS: The systemic inflammatory response syndrome; Th: T-helper cell.

## Competing interests

There are no competing interests to declare.

## Authors’ contributions

Study design: Y-SC, W-JK, L-PC, T-HH. Data collection: T-HH, C-TH, Y-SC. Statistical analysis: C-HC, C-FL, H-HL. Interpretation: T-HH, Y-SC, W-JK, S-LY, L-PC, C-TH. Manuscript preparation: T-HH, Y-SC, W-JK, C-HC. All authors read and approved the final manuscript.
